# Comparative Analysis of Mandibular Kinematics Recorded with Zebris JMA and Medit i700 Using an Open-Source 3D Model-Based Framework

**DOI:** 10.3390/dj14070455

**Published:** 2026-07-20

**Authors:** Radu-Gabriel Ionescu, Alexia-Ecaterina Cârstea, Vlad-Gabriel Vasilescu, Florin-Octavian Froimovici, Tudor-Claudiu Spînu, Lucian-Toma Ciocan, Camelia Ionescu

**Affiliations:** 1 Faculty of Dentistry, “Carol Davila” University of Medicine and Pharmacy, Dionisie Lupu Street, No. 37, District 2, 020021 Bucharest, Romania; radu-gabriel.ionescu0720@stud.umfcd.ro; 2 Department of Dental Prostheses Technology, Faculty of Dentistry, “Carol Davila” University of Medicine and Pharmacy, 37 Dionisie Lupu Street, District 2, 020021 Bucharest, Romania; florin-octavian.froimovici@drd.umfcd.ro (F.-O.F.); lucian.ciocan@umfcd.ro (L.-T.C.); camelia.ionescu@umfcd.ro (C.I.); 3 Department of Prosthodontics, Faculty of Dentistry, “Carol Davila” University of Medicine and Pharmacy, 37 Dionisie Lupu Street, District 2, 020021 Bucharest, Romania; tudor.spinu@umfcd.ro

**Keywords:** mandibular kinematics, jaw motion analysis, digital dental avatar, Zebris JMA, Medit i700, Blender4Dental, intraoral scanning

## Abstract

**Background/Objectives:** Mandibular kinematics are increasingly central to the construction of the digital dental avatar, and can be recorded with two categories of devices: dedicated jaw motion analysers and intraoral scanners with motion-tracking modules. Few studies have compared these categories on the same patients. This pilot study compared the Zebris JMA and Medit i700 in a paired-cohort design, using an open-source Blender-based framework developed for the uniform analysis of their XML exports. **Methods:** A prospective, observational, paired-cohort study was conducted on 17 adults recorded with both systems by a single operator. A custom Python 3.11 add-on for Blender 4.5 LTS and Blender4Dental 1.1.99, developed within the study, supported the analytical workflow. A common anatomical origin between the two systems was first validated by comparing the occlusal contact area at MIP. At each threshold, the two systems were then compared on the directional decomposition (X, Y, Z components) of the mandibular displacement along the anatomical axes. Agreement was evaluated using paired *t*-tests or Wilcoxon signed-rank tests, Bland–Altman analysis, Pearson or Spearman correlation coefficients, and the intraclass correlation coefficient. **Results:** Validation at maximum intercuspation showed near-perfect agreement between the two workflows (Pearson r = 0.997; ICC(2,1) = 0.997). During protrusion, the dominant displacement component shifted to the anteroposterior axis. SVD residuals were smaller for Medit (~0.003–0.004 mm) than for Zebris (~0.024–0.046 mm; *p* < 0.001), but were interpreted only as internal rigid-body fitting residuals rather than accuracy metrics. **Conclusions:** Zebris JMA and Medit i700 yielded similar scalar displacement values under the threshold-based frame-selection procedure, but showed systematic differences in directional decomposition.

## 1. Introduction

Digital technologies for acquisition, design and manufacturing have become increasingly integral to prosthetic and restorative dentistry over the past decade. Intraoral scanning, cone-beam computed tomography (CBCT), facial scanning, mandibular kinematics recording, and computer-aided design and manufacturing (CAD/CAM) now form a coherent clinical workflow, and their combined use has become routine in contemporary prosthodontic practice [1,2,3]. One of the most significant outcomes of this evolution is the digital dental avatar: a patient-specific virtual construct that reproduces both the morphological characteristics of the dentition and supporting structures and the dynamic behavior of the oral and maxillofacial system [4,5,6]. Realizing this construct requires the acquisition and integration of three-dimensional surface meshes from intraoral scanners, volumetric reconstructions from CBCT, and time-resolved kinematic records from jaw motion analyzers into a single, clinically actionable representation [5,7,8,9,10]. When the temporal axis of mandibular kinematics is superimposed on the static anatomy, the resulting construct is commonly referred to as the four-dimensional (4D) virtual patient and is increasingly identified as the integration target for contemporary digital workflows [11,12,13,14,15].

Of the various components that contribute to the 4D virtual patient, mandibular kinematics is among the least standardized across clinical platforms. Acquisition systems fall into two broad technological families. The first comprises dedicated jaw motion analyzers, including the electromagnetic Cadiax 4 (GAMMA Medizinisch-wissenschaftliche Fortbildungs-GmbH, Klosterneuburg, Austria), long established as a reference standard in computerized condylography; the ultrasound- and infrared-based Zebris JMA (Zebris Medical GmbH, Isny im Allgäu, Germany); the high-speed infrared MODJAW (MODJAW SAS, Villeurbanne, France); and the high-precision camera Cyclops platform (ITAKA WAY MED Srl, Marcon, Italy) [16,17,18,19,20,21,22]. The second, more recent, comprises intraoral scanners equipped with native motion-tracking modules designed to reduce chair-side time, exemplified clinically by the Medit i700 (Medit Corp., Seoul, Republic of Korea) and by the Shining Aoralscan (SHINING 3D Tech Co., Ltd., Hangzhou, China) [23,24,25,26]. The two families have different acquisition principles. Dedicated analyzers reconstruct mandibular trajectories with reference to a head-mounted cranial frame and an externally tracked marker assembly, whereas intraoral-scanner-based modules derive displacement directly from the geometric correspondence of successive intraoral surface scans, without recourse to any head-fixed referential. The clinical and interpretive consequences of this divergence have received comparatively little attention in the published literature.

A growing body of evidence indicates that inter-system variability in functional dental acquisition is also clinically meaningful. Vlăduțu et al., working with paired Medit i700 recordings processed through two analytical environments (Medit Occlusion Analyzer and Exocad 3.2), reported significantly divergent occlusal contact distributions for identical raw data, with the disagreement largest in dynamic positions [24,25]. That finding isolates the software component of inter-system variability and shows that the analytical pipeline can affect clinical interpretation even when the acquisition substrate is held constant. At the hardware level, two recent paired-cohort investigations have shown that Cadiax and MODJAW yield systematically different values of the Bennett angle and sagittal condylar inclination, particularly at small mandibular amplitudes, and have concluded that the two systems are not interchangeable for articulator programming despite their nominal similarity in clinical practice [17,18]. Pairwise comparisons between Zebris JMA and Cadiax [27] and between MODJAW and Cadiax [17] round out the published comparative landscape for dedicated analysers. To the authors’ knowledge, direct paired clinical comparisons between dedicated jaw motion analyzers and intraoral-scanner-based motion modules remain limited, particularly when both systems are applied to the same participants and analyzed on a common anatomical geometry. Whether the two categories yield comparable representations of mandibular kinematics therefore remains an open question.

A second consideration concerns the tools available for processing kinematic data once acquired. Proprietary CAD environments such as Exocad 3.2 (exocad GmbH, Darmstadt, Germany) and Planmeca CAD Premium 3.0 (Planmeca Oy, Helsinki, Finland) accept XML import from jaw motion analyzers and provide animated playback, but as closed-source platforms, they offer limited access to the per-frame translational and rotational decomposition, do not readily support threshold-based frame selection, and do not easily allow the export of the underlying numerical kinematics for independent analysis [16,28,29,30,31,32]. The open-source Blender4Dental 1.1.99 extension, developed on Blender 4.5 LTS (Blender Foundation, Amsterdam, The Netherlands), provides a transparent and extensible dental modeling environment that includes a virtual articulator module; in its current release, it does not natively allow the import of XML files from commercial motion analyzers and operates instead from manually entered Bennett and condylar inclination parameters, which by construction cannot reproduce patient-specific kinematics. The closest related open-source contribution, reported by Ruggiero and colleagues, targets the acquisition end of the workflow using consumer-grade RGB-D sensors and does not address the downstream analysis of XML exports from commercial systems [10,21,33,34]. A review of the currently available solutions indicates that no widely available open, transparent, frame-aware platform provides this combination of functions for XML kinematic data: vendor-specific import, frame-level reconstruction, threshold-based selection, numerical export, and contact-area analysis on patient-specific dental meshes [27,35,36,37]. This practical gap limits independent interrogation of commercial jaw-motion exports and complicates direct head-to-head comparisons between acquisition systems.

Accordingly, this pilot methodological study had two complementary aims. The primary aim was to develop and document an open-source Blender-based workflow for importing, reconstructing, and analysing XML mandibular-motion exports from Zebris JMA and Medit i700 within a common three-dimensional environment. The secondary aim was to apply this workflow in a paired cohort of 17 adults to explore inter-system agreement in total displacement magnitude and directional vector decomposition during standardized mandibular excursions. The study was intended to evaluate their agreement and system-specific reconstruction behaviour under identical paired clinical conditions, using the same anatomical STL/OBJ/PLY geometry and standardized displacement thresholds from maximum intercuspation [38,39,40].

A common functional origin between the two systems was first assessed by comparing the occlusal contact area at maximum intercuspation. The null hypothesis was that no systematic inter-system differences would be observed in the directional decomposition of mandibular displacement when both systems were applied to the same participants and analysed against a common geometric reference.

## 2. Materials and Methods

### 2.1. Study Design

This investigation was designed as a prospective, observational, paired-cohort comparative pilot study. A within-subject design was selected because each participant was recorded with both acquisition systems, allowing direct comparison of the Zebris JMA and Medit i700 under the same anatomical and functional conditions. This design was intended to reduce the influence of inter-individual variability in occlusal morphology, mandibular range of motion, neuromuscular coordination, and temporomandibular joint anatomy.

**Figure 1:**
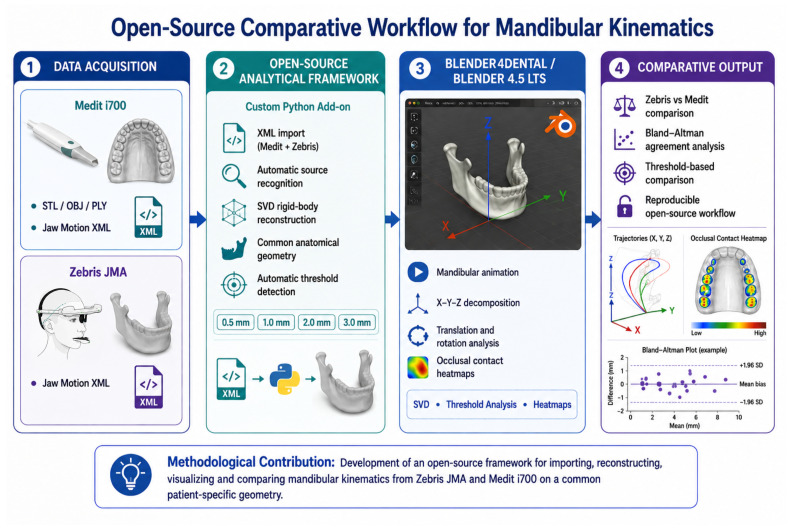
Open-source workflow for comparative analysis of mandibular kinematics.

The study was conducted at the Department of Prosthodontics, Faculty of Dental Medicine, “Carol Davila” University of Medicine and Pharmacy, Bucharest, Romania. Ethical approval was obtained from “Carol Davila” University of Medicine and Pharmacy, Bucharest, Romania (Approval No. 11716/8 May 2026). The study was conducted in accordance with the Declaration of Helsinki. All participants provided written informed consent before enrolment after receiving verbal and written information regarding the non-invasive recording protocol, the expected duration of the session, and their right to withdraw at any time without consequence. The overall open-source workflow used for the comparative analysis of mandibular kinematics is summarized in Figure 1. Unless otherwise specified, workflow figures are schematic representations created to illustrate the methodological sequence used in the study. They are not intended to reproduce proprietary device interfaces, commercial software screens, or manufacturer-specific hardware configurations.

### 2.2. Participants

Seventeen adult participants were prospectively recruited at the Department of Prosthodontics in May 2026. Each participant underwent mandibular movement recording with both acquisition systems, Zebris JMA and Medit i700, thereby providing paired within-subject data for the comparative analysis. This design ensured that both systems were evaluated on the same occlusal morphology, mandibular range of motion, and functional movement pattern.

Participants were eligible for inclusion if they presented a functional dentition in both arches, sufficient to support a stable and reproducible maximum intercuspation position (MIP), and if they were able to perform left laterotrusion, right laterotrusion, and protrusion to an edge-to-edge position. Additional inclusion criteria were tolerance of the paraocclusal mandibular fork required for Zebris JMA acquisition and willingness to complete both recording sessions.

Exclusion criteria included significant limitation of mouth opening, unstable prosthetic restorations that could interfere with rigid fixation of the mandibular paraocclusal fork, recent occlusal adjustment, documented neuromuscular pathology affecting mandibular function, inability to maintain a reproducible MIP, or acquisition errors that prevented paired analysis of the corresponding movement.

The final analytical cohort included 9 females and 8 males, with a mean age of 26.5 ± 6.4 years (range: 22–50 years). Regarding occlusal classification, 10 participants presented Angle Class I occlusion, 5 presented Angle Class II occlusion, and 2 presented Angle Class III occlusion. One participant classified as Angle Class I also presented an anterior open bite. Detailed subgroup analyses according to temporomandibular disorder symptoms, restorative/prosthetic status, or mandibular range of motion were not used as stratification factors in this pilot methodological study and are acknowledged as limitations for broader clinical generalization.

### 2.3. Acquisition Protocols

All acquisitions were performed by a single calibrated operator according to a standardised clinical workflow. The acquisition sequence was maintained constant for all participants in order to minimise protocol-related variability and to preserve the integrity of the anatomical reference used for the comparative analysis.

The workflow began with static intraoral scanning using the Medit i700. Maxillary and mandibular STL/OBJ/PLY models were acquired and used as the common anatomical geometry for both acquisition systems.

After acquisition of the static STL/OBJ/PLY models, mandibular motion was recorded using the Medit i700 jaw motion module. Each participant was instructed to start from maximum intercuspation position (MIP) and to perform right laterotrusion, left laterotrusion, and protrusion to an edge-to-edge position. These recordings formed the intraoral-scanner-based dynamic dataset used for subsequent analysis.

**Figure 2:**
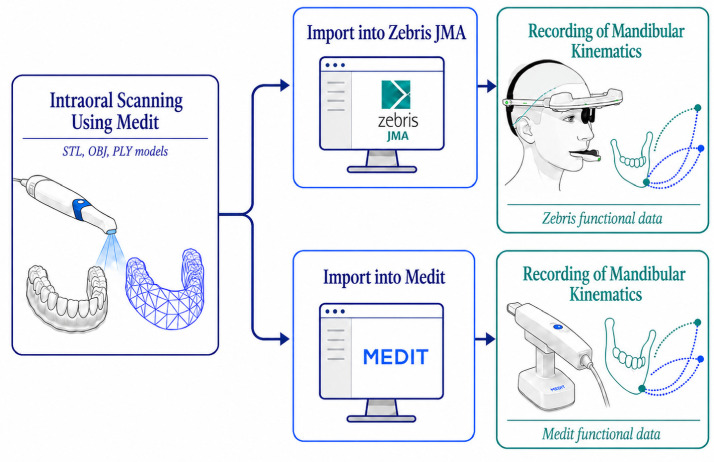
General acquisition scheme for static morphology and mandibular kinematics. Static dental morphology was acquired by intraoral scanning with the Medit system, generating patient-specific surface models in STL, OBJ, or PLY format.

The Zebris JMA recording was performed subsequently. The same mandibular movements were recorded: right laterotrusion, left laterotrusion, and protrusion to an edge-to-edge position, each initiated from MIP. The Zebris protocol required adaptation and rigid fixation of a mandibular paraocclusal fork to the lower dental arch, as described in Section 2.3.2. The resulting XML files provided the dedicated jaw-motion-analyser dataset. The general acquisition sequence, including static intraoral scanning and the two dynamic recording pathways, is illustrated in Figure 2.

For both systems, MIP was considered the functional origin of mandibular movement. Because Zebris JMA and Medit i700 export kinematic data within independent coordinate systems, the raw marker coordinates of the two systems were not compared directly. Instead, mandibular displacement was calculated relative to the MIP reference of each system. Inter-system comparisons were therefore performed at standardised displacement thresholds of 0.5, 1.0, 2.0, and 3.0 mm from the initial intercuspal position. These thresholds were anchored in the existing occlusion and mandibular-movement literature and were selected to represent clinically meaningful phases of dynamic occlusion, ranging from the MIP-adjacent transition zone to edge-to-edge functional excursion [38,39,40].

This relative, threshold-based approach allowed both systems to be evaluated at similar functional amplitudes while using the same anatomical STL/OBJ/PLY geometry. The comparison was designed to determine whether the two systems reconstruct mandibular motion similarly when the mandible has reached the same displacement from MIP, rather than to search for isolated frames in which comparable occlusal contacts might occur under different translational or rotational configurations.

#### 2.3.1. Medit i700 Dynamic Recording Protocol

Dynamic mandibular motion was acquired using the Medit i700 intraoral scanner (Medit Corp., Seoul, Republic of Korea) and the jaw motion module integrated in Medit Link 3.4.9 [24,25,27]. Prior to acquisition, the scanner was calibrated in accordance with the manufacturer’s instructions.

For each participant, three functional mandibular excursions were recorded: right laterotrusion, left laterotrusion, and protrusion. Each movement cycle was initiated from maximum intercuspation position, continued until the corresponding edge-to-edge endpoint was reached, and then returned to maximum intercuspation. After returning to MIP, the participant was instructed to maintain the intercuspal position for approximately 2–3 s before repeating the same movement cycle.

**Figure 3:**
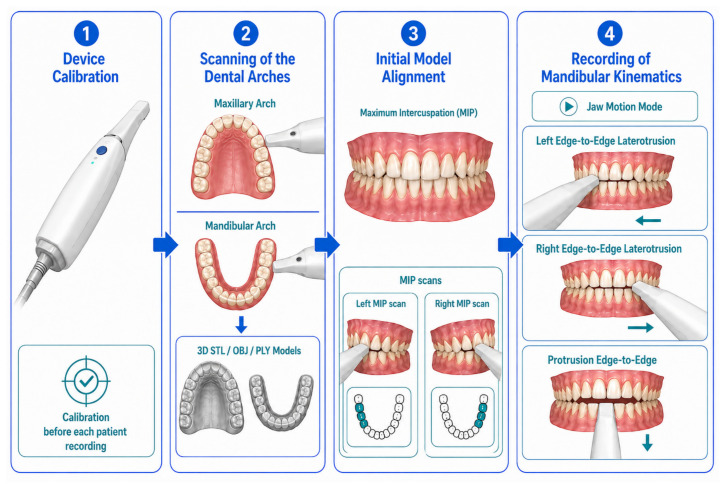
Workflow for intraoral scanning and mandibular kinematics recording with the Medit i700 system.

Scanner positioning and movement acquisition were performed according to the manufacturer’s recommendations. Throughout acquisition, particular attention was directed toward maintaining uninterrupted optical tracking of the dental surfaces. Recordings affected by loss of tracking, loss of focus, incomplete excursion, or an inconsistent starting position were not accepted for analysis. The Medit i700 scanning and jaw-motion acquisition workflow is presented in Figure 3.

Each movement was recorded over multiple cycles in order to verify technical consistency and to obtain a recording that fulfilled the predefined acquisition criteria. For the final analysis, the technically acceptable cycle that best satisfied these criteria was selected for each movement. The dynamic data were exported as XML files containing movement-specific tracking information and the initial scan position used by the software as the reference configuration. The native sampling frequency of the Medit recordings ranged from 16 to 23 Hz, depending on tracking conditions during acquisition, and was preserved during export for subsequent synchronisation in Blender.

#### 2.3.2. Zebris JMA Dynamic Recording Protocol

Mandibular kinematics were acquired using the Zebris JMA system (Zebris Medical GmbH, Isny im Allgäu, Germany) with Tizian Function Pro 3.0.2 software (Schütz Dental GmbH, Rosbach vor der Höhe, Germany). The system records mandibular movement by tracking an infrared marker assembly attached to the mandibular paraocclusal fork, which is detected by a head-mounted facial arc containing four infrared sensors [17,18].

At the beginning of the recording session, the facial arc was positioned on the participant’s head, with the four sensors oriented toward the perioral region. Cranial orientation was then performed using the C-bow carrying the reference marker. The anatomical landmarks required for orientation, including the left Porion, right Porion, and Orbitale, were identified. These landmarks were used to establish the Frankfurt horizontal plane as the patient-specific cranial reference plane [41].

After cranial orientation, the maxillary alignment fork carrying its marker was positioned on the maxillary arch. This step registered the fixed spatial position of the maxilla within the Zebris coordinate system and established the static maxillary reference for subsequent mandibular movement recording.

The mandibular paraocclusal fork was then individually adapted to the lower dental arch and rigidly fixed to the mandibular teeth using self-polymerising bis-acrylic composite resin (Structur 3, Voco, Cuxhaven, Germany). The mandibular fork carried the infrared marker assembly used for dynamic tracking. Its position was detected by the four sensors of the facial arc throughout the movement, allowing the three-dimensional mandibular trajectory to be recorded relative to the previously established cranio-maxillary reference.

The same functional excursions recorded with the Medit system were acquired with Zebris JMA: right laterotrusion, left laterotrusion, and protrusion. Each movement cycle was initiated from maximum intercuspation position, continued to the corresponding edge-to-edge endpoint, and then returned to maximum intercuspation. Movements were repeated when necessary to ensure a technically valid acquisition and to exclude recordings affected by incomplete excursions, inconsistent starting position, or movement artefacts.

**Figure 4:**
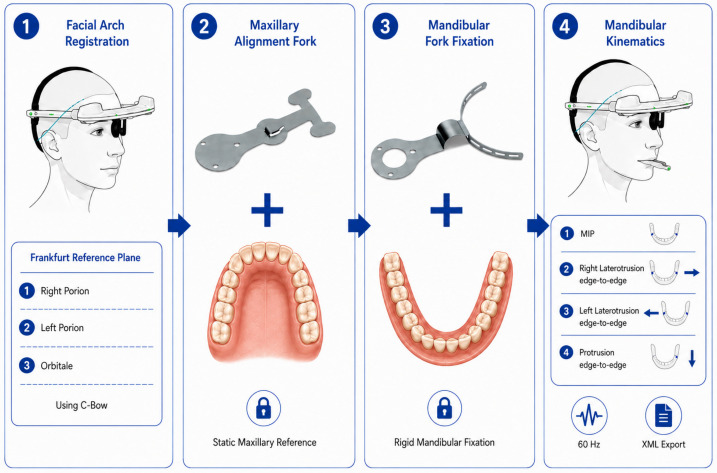
Workflow for mandibular kinematics acquisition with the Zebris JMA system.

For both acquisition systems, repeated movement cycles were recorded when necessary until a technically valid recording was obtained. A cycle was considered technically valid when it showed a stable MIP starting position, uninterrupted tracking, completion of the intended excursion, absence of obvious movement artefacts, and a clinically plausible return trajectory. Cycle selection was based on these technical validity criteria and on visual continuity of the recorded movement before statistical comparison. It was not based on the magnitude or direction of the subsequent inter-system differences. The number of repeated acquisition attempts was not systematically logged, and intra-session repeatability was not quantified in the present pilot study. The internal visual verification of the reconstructed motion across the available software environments is described separately in Section 2.4.1. The Zebris JMA acquisition workflow, including cranial orientation, fork registration, and movement recording, is presented in Figure 4.

Kinematic data were recorded at 60 Hz and exported as XML files. The exported data contained the static intercuspal reference configuration, identified in the XML structure as reference_ikp, and the time-resolved three-dimensional coordinates of the mandibular tracking markers for each recorded movement.

#### 2.3.3. Common STL/OBJ/PLY Geometry

The static maxillary and mandibular STL/OBJ/PLY models obtained with the Medit i700 were used as the common anatomical geometry for both systems. In the analytical workflow, these models were manipulated as rigid digital bodies, meaning that their mesh geometry was not modified during animation or contact-area computation.

The same mandibular OBJ model was animated using either the Medit-derived or the Zebris-derived kinematic transformations. This approach isolated the kinematic reconstruction method as the main variable of interest and reduced confounding effects related to surface morphology, mesh density, or interarch model alignment.

### 2.4. Open-Source Analytical Framework

A custom Python 3.11 add-on, designated JMA XML Animator v1.0.0, was developed to provide a transparent, frame-aware workflow for the analysis of mandibular kinematic data exported by commercial acquisition systems. The add-on was implemented for Blender 4.5 LTS and Blender4Dental 1.1.99 and was designed to import, visualise, and quantify mandibular motion from XML files generated by both the Medit i700 and Zebris JMA systems.

The development of this framework was intended to address a specific methodological gap in the current digital dentistry software ecosystem: the lack of a transparent workflow for importing, reconstructing, and quantitatively analysing vendor-specific XML mandibular-motion exports on patient-specific 3D dental models. Commercial CAD/CAM platforms, such as Exocad 3.2 and Planmeca CAD Premium 3.0, can visualise mandibular motion and may support the import of jaw-motion records, but they generally provide limited access to the underlying frame-by-frame kinematic data, displacement components, contact area, and threshold-specific positions [16,28,29]. Conversely, Blender4Dental 1.1.99 provides an extensible 3D modelling environment and virtual articulator tools, but it does not natively import XML motion files from commercial jaw-tracking systems or reconstruct patient-specific mandibular trajectories directly from such data [33].

The proposed add-on was therefore developed as a bridge between commercial kinematic acquisition systems and open-source three-dimensional analysis of patient-specific dental meshes. It automatically parses XML files exported by Medit and Zebris, reconstructs mandibular motion using rigid-body transformations, animates the mandibular OBJ model within Blender, identifies clinically relevant displacement thresholds, and enables geometric analysis of occlusal contact areas on the same anatomical models.

**Figure 5:**
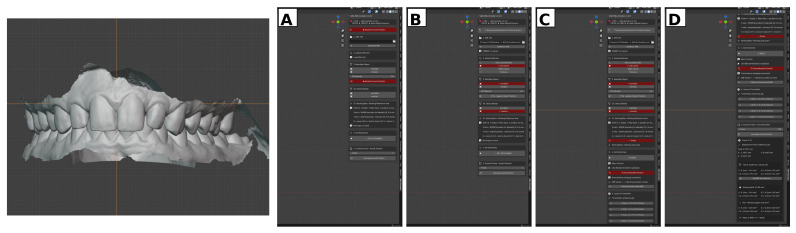
Architecture and workflow of the JMA XML Animator v1.0.0 add-on for Blender 4.5 LTS and Blender4Dental 1.1.99. (**A**) XML and 3D model import. (**B**) Movement selection. (**C**) Animation and threshold-based frame selection. (**D**) Contact-area analysis and output.

The methodological contribution of the framework lies in its ability to transform vendor-specific kinematic exports into an inspectable and reproducible analytical workflow. Unlike conventional visualisation-only approaches, the add-on allows frame-by-frame inspection, quantification of displacement relative to the initial intercuspal position, comparison of kinematic reconstructions from different systems on the same OBJ geometry, and linkage of dynamic mandibular position to occlusal contact distribution. The architecture and operational workflow of the JMA XML Animator v1.0.0 add-on are shown in Figure 5.

#### 2.4.1. Automatic XML Format Detection and Parsing

After loading an XML file, the add-on automatically identified the acquisition system by inspecting system-specific reference elements. Zebris JMA files were recognised by the presence of reference_ikp, whereas Medit i700 files were recognised by the presence of scan_position. For each movement, the XML file contained three marker objects, each defined by x, y, and z coordinates at every recorded frame. The parser extracted the available movement, the native acquisition frequency, and the frame-by-frame coordinates of these three markers. The extracted data were converted into a common internal structure, allowing the same analytical procedures such as SVD transformation, displacement calculation, threshold selection, animation, and contact-area computation to be applied to both systems.

Mandibular motion was reconstructed within each system from the relative change between the reference configuration at maximum intercuspation and each subsequent frame, as detailed mathematically in Section 2.4.2.

Internal workflow verification was performed as a qualitative visual consistency check between the reconstructed motion in Blender/Blender4Dental and the corresponding visualisations in the native or compatible software environments. Medit-derived recordings were checked against Medit Occlusion Analyzer 1.0.2 (Medit Corp., Seoul, Republic of Korea) and Planmeca CAD Premium 3.0, while Zebris-derived recordings were checked against Tizian Function Pro 3.0.2 and Planmeca CAD Premium 3.0. This check was performed at maximum intercuspation and at the movement endpoint defined operationally by the instructed edge-to-edge relationship, using common colour-scale heatmaps of the distance between the mandibular and maxillary meshes. In addition, the gross trajectory pattern was visually inspected to confirm that the reconstructed motion followed the expected movement sequence from maximum intercuspation toward the instructed edge-to-edge endpoint and back, without obvious reversal, axis inversion, or discontinuity. The purpose of this step was to verify visual consistency of the exported XML reconstruction within the study workflow, not to provide an independent numerical validation of acquisition accuracy. Consequently, this verification step was used only to confirm that the imported XML motion was reconstructed in the expected direction and endpoint configuration before quantitative analysis was performed.

#### 2.4.2. Rigid-Body Transformation Using Singular Value Decomposition

Mandibular movement was reconstructed as a sequence of rigid-body transformations. The method is based on the classical least-squares registration of two corresponding three-dimensional point sets, in which the objective is to estimate the rotation and translation that best align one marker configuration with another without deforming the object [42,43].

For each recorded frame, the reference marker configuration was defined as:

(1) $$A=\left[\begin{array}{ccc} x_{1,A} & y_{1,A} & z_{1,A}\\ x_{2,A} & y_{2,A} & z_{2,A}\\ x_{3,A} & y_{3,A} & z_{3,A} \end{array}\right],$$

and the current marker configuration as:

(2) $$B_{i}=\left[\begin{array}{ccc} x_{1,i} & y_{1,i} & z_{1,i}\\ x_{2,i} & y_{2,i} & z_{2,i}\\ x_{3,i} & y_{3,i} & z_{3,i} \end{array}\right],$$

where each row corresponds to one of the three XML markers, mark_1, mark_2, and mark_3.

The centroids of the two configurations were calculated as:

(3) $$c_{A}=\frac{1}{3}\sum_{k=1}^{3}A_{k},c_{B_{i}}=\frac{1}{3}\sum_{k=1}^{3}B_{i,k}$$

The centred point sets were then obtained by subtracting their respective centroids:

(4) $$\tilde{A}=A-c_{A},\tilde{B_{i}}=B_{i}-c_{B_{i}}$$

The cross-covariance matrix was calculated as:

(5) $$H_{i}={\tilde{A}}^{T}\tilde{B_{i}}$$

Singular value decomposition was then applied to $H_{i}$:

(6) $$H_{i}=U_{i}\Sigma_{i}V_{i}^{T}$$

The optimal rotation matrix was obtained as:

(7) $$R_{i}=V_{i}U_{i}^{T}$$

If $det(R_{i})<0$, a reflection correction was applied by changing the sign of the last row of $V_{i}^{T}$, after which $R_{i}$ was recomputed.

The translation vector was calculated as:

(8) $$t_{i}=c_{B_{i}}-R_{i}c_{A}$$

Finally, the mandibular displacement magnitude at frame i was defined as the Euclidean norm of the translation vector:

(9) $$d_{i}=∥t_{i}∥=\sqrt{t_{x,i}^{2}+t_{y,i}^{2}+t_{z,i}^{2}}$$

This procedure was implemented in Python 3.11 using NumPy. In the add-on, the rigid_transform (A, B) function calculates the centroids, constructs the cross-covariance matrix, applies np.linalg.svd, performs determinant-based reflection correction, and returns the rotation matrix $R_{i}$ and translation vector $t_{i}$. The same transformation was used to animate the mandibular OBJ model in Blender and to compute the displacement magnitude used for threshold selection.

#### 2.4.3. Blender Timeline Synchronisation and Mandibular Animation

After the rigid-body transformation was calculated for each recorded frame, the resulting rotation matrix and translation vector were converted into Blender-compatible transformations and applied to the mandibular OBJ object. The maxillary OBJ model remained unchanged, while the mandibular OBJ model was animated as a rigid digital object.

The exported acquisition frequency was used only for temporal mapping of XML frame indices to Blender timeline frames during animation playback. This temporal mapping affected only the position of keyframes on the Blender timeline and did not modify the exported three-dimensional coordinates, the SVD-derived rigid-body transformations, or the displacement components used for threshold selection and statistical analysis. Threshold-based frame selection is described separately in Section 2.4.4.

This procedure generated a complete patient-specific mandibular animation for each recorded movement. The resulting animation could be visualised frame by frame within Blender and Blender4Dental, allowing direct inspection of the reconstructed trajectory before threshold-based frame selection and occlusal contact-area analysis.

#### 2.4.4. Threshold-Based Frame Selection

After the displacement magnitude had been calculated for each recorded frame, the add-on identified the frames corresponding to the predefined displacement thresholds of 0.5, 1.0, 2.0, and 3.0 mm from the initial intercuspal position.

For each threshold, the displacement magnitude of every frame was compared with the target value. The selected frame was the recorded frame with the smallest absolute difference between the measured displacement and the target threshold:

$$i_{\tau }=arg\underset{i}{min}|d_{i}-\tau |,$$

where $d_{i}$ is the displacement magnitude at frame i, and τ represents the target threshold.

This nearest-frame approach was used because the two systems had different native acquisition frequencies and did not necessarily contain a frame at the exact target displacement. If a movement did not reach a specific threshold, that threshold was flagged as unavailable and excluded from the corresponding threshold-specific paired analysis. Threshold selection was therefore based on geometric displacement relative to maximum intercuspation, not on elapsed time or movement speed.

The selected threshold frames were then used for subsequent comparison of mandibular position and occlusal contact-area computation. This ensured that Medit i700 and Zebris JMA recordings were compared at comparable scalar displacement amplitudes relative to their own MIP reference positions. Because different sampling frequencies may theoretically affect the availability of frames close to a desired target threshold, the absolute deviation between the selected frame and each threshold was quantified and reported in Supplementary Table S1 with the aggregated values per system reported in Supplementary Table S2.

#### 2.4.5. Occlusal Contact Area Computation

Occlusal contact area was computed geometrically from the mesh data using the contact-area module implemented in the add-on. For each analysed frame, the mandibular mesh was positioned according to the corresponding rigid-body transformation. A KD-tree was then constructed from the transformed mandibular vertices, and the minimum distance from each maxillary vertex to the mandibular mesh was calculated.

For each maxillary triangle, the mean distance of its three vertices to the transformed mandibular mesh was used to assign the triangle to one of four proximity intervals: 0.0–0.1 mm, 0.1–0.2 mm, 0.2–0.3 mm, and 0.3–0.5 mm. Triangles with a mean distance of 0.5 mm or greater were excluded from the contact-area calculation. The area of each triangle was calculated in world coordinates and summed within the corresponding proximity interval, producing contact-area values in square millimetres (mm^2^) [24,25,38].

In the present analysis, the MIP contact-area comparison was used as a validation step to verify that the two systems were anchored to a comparable functional origin before dynamic kinematic comparisons were interpreted. The same computational framework also supports contact-area calculation at selected dynamic threshold frames, which represents a further analytical extension of the workflow.

#### 2.4.6. Working- and Non-Working-Side Separation

For laterotrusive movements, occlusal contact areas were further classified according to working and non-working side [40]. A midline reference was defined within the Blender scene, and the centroid of each maxillary mesh triangle was used to assign the corresponding contact area to one side of the arch.

During right laterotrusion, contacts located on the patient’s right side were classified as working-side contacts, whereas contacts on the left side were classified as non-working-side contacts. The reverse convention was applied for left laterotrusion. This classification allowed the contact-area output to be interpreted according to the functional side of mandibular movement.

For protrusion, the same principle was adapted to distinguish anterior guidance contacts from posterior residual contacts or potential interferences. This side-based classification was used to support the clinical interpretation of the contact-area distribution generated by each kinematic reconstruction.

This side-based classification is implemented as an available output of the framework and was not analysed quantitatively in the present study. The current analysis was focused on the inter-system comparison of displacement decomposition and total occlusal contact area at MIP; quantitative side-specific contact analysis is planned as a follow-up study using the same open-source framework.

#### 2.4.7. Integration with Blender4Dental

The add-on was designed to operate within the Blender environment while remaining compatible with Blender4Dental 1.1.99. After the XML file was parsed and the mandibular keyframes were generated, the resulting animation could be inspected directly in Blender and used together with the Blender4Dental articulator and visualisation modules.

In this workflow, the add-on functioned as an upstream kinematic pre-processor. It imported the motion data, reconstructed the mandibular trajectory, and applied the corresponding keyframes to the mandibular OBJ object. Blender4Dental was then used as the visual environment for playback, frame inspection, and qualitative heatmap visualisation.

This integration allowed patient-specific mandibular movement derived from Medit i700 or Zebris JMA XML files to be analysed within a single open 3D workspace, without modifying the core Blender4Dental software.

### 2.5. Statistical Analysis

All statistical analyses were performed in IBM SPSS Statistics version 29.0.2.0 (IBM Corp., Armonk, NY, USA). Prior to inferential testing, the normality of the paired within-subject differences (Zebris–Medit) was assessed using the Shapiro–Wilk test, which is appropriate for small sample sizes [44].

Descriptive statistics are reported as mean ± standard deviation (SD), together with the corresponding 95% confidence intervals for paired differences. For paired comparisons in which the differences satisfied the normality assumption (Shapiro–Wilk *p* > 0.05), the paired-samples *t*-test was applied, with Cohen’s $d_{z}$ reported as the standardised effect size [45]:

$$d_{z}=\frac{\bar{D}}{SD_{D}},$$

where $\bar{D}$ is the mean paired difference and $SD_{D}$ is the standard deviation of the paired differences. Effect-size thresholds were interpreted as 0.20 small, 0.50 medium, and 0.80 large.

For paired comparisons in which the differences departed significantly from normality (Shapiro–Wilk *p* ≤ 0.05), the non-parametric Wilcoxon signed-rank test was applied, with the effect size reported ass [46]:

$$r=\frac{|Z|}{\sqrt{N}},$$

where Z is the standardised Wilcoxon statistic and N is the number of valid paired observations. Effect-size thresholds were interpreted as 0.10 small, 0.30 medium, and 0.50 large.

Agreement between the two acquisition systems was further characterised using Bland–Altman analysis [47,48,49], reporting the mean bias and the 95% limits of agreement:

$$LoA=bias\pm 1.96\times SD_{diff}.$$

Bland–Altman bias and 95% limits of agreement were considered the primary agreement metrics. Association between systems was quantified using Pearson’s product–moment correlation coefficient (r) for parametric pairs or Spearman’s rank correlation coefficient (ρ) for non-parametric pairs. Correlation coefficients were reported only as descriptive measures of association and were not interpreted as evidence of agreement or interchangeability. For the MIP contact-area validation, the intraclass correlation coefficient was calculated as ICC(2,1), corresponding to a two-way random-effects model, absolute agreement, single measures, with 95% confidence interval [50,51]. This ICC model was selected because the analysis focused on absolute agreement between the two acquisition workflows rather than on consistency alone.

To provide a clinical reference framework for the Bland–Altman analysis, a clinical reference margin of ±0.5 mm was adopted for inter-system disagreement, consistent with the conventional threshold of clinical relevance in occlusal measurement and with the lowest proximity-interval boundary used in our contact-area computation.

For component-wise outcomes, inferential testing was performed separately for each movement × threshold condition, so that each participant contributed at most one paired observation per condition. Aggregated values across thresholds were used only descriptively and were not treated as independent observations for inferential testing.

Statistical significance was set at α=0.05, two-tailed. Missing observations, corresponding to trials in which one system did not reach the requested displacement threshold, were excluded pairwise. The number of valid paired observations (n) is reported separately for each test. Ninety-five percent confidence intervals were reported for the ICC, paired mean differences, Bland–Altman bias, and limits of agreement.

## 3. Results

### 3.1. Sample Characteristics and Data Completeness

For left laterotrusion, one participant did not generate valid Zebris recordings across the first three displacement thresholds, and two participants were unavailable for Zebris analysis at the 3.0 mm threshold. For right laterotrusion, one participant was unavailable for Zebris analysis across all thresholds. For protrusion, all participants contributed valid recordings up to the 2.0 mm threshold, while two Zebris recordings were unavailable at the 3.0 mm threshold. For Medit i700, all participants reached the 0.5, 1.0, and 2.0 mm thresholds across all movements, while one recording was unavailable at the 3.0 mm threshold for left laterotrusion and one for right laterotrusion.

These exclusions are reflected in the threshold-specific sample sizes reported throughout the tables, with n ranging from 14 to 17 per condition. Acquisition success rates by system, movement, and threshold are reported in Table 1.

The Zebris system reached an overall success rate of 193/204 recordings (94.6%) across all eligible system-specific recordings. The Medit system reached an overall success rate of 202/204 recordings (99.0%), with failures observed exclusively at the 3.0 mm threshold. Threshold-specific paired comparison using McNemar’s exact test for the proportion of paired successes per condition did not reach statistical significance for any movement × threshold combination (all *p* ≥ 0.500), indicating no statistically detectable difference in acquisition feasibility between the two systems within the present pilot sample.

### 3.2. Assessment of the Common Functional Origin at Maximum Intercuspation

Before analysing dynamic mandibular displacement, the comparability of the functional origin of the two kinematic reconstructions was assessed at maximum intercuspation. Total occlusal contact area at MIP was calculated on the common OBJ geometry using the predefined contact-proximity range up to 0.5 mm and was compared between the Zebris-derived and Medit-derived reconstructions.

The mean total contact area was 280.94 ± 130.29 mm^2^ for Zebris and 283.82 ± 129.40 mm^2^ for Medit. The mean paired difference was +2.88 mm^2^, corresponding to a mean absolute percentage difference of 2.04%. The two workflows showed close numerical correspondence at MIP. Pearson correlation was r = 0.997 (*p* < 0.001), and the intraclass correlation coefficient was ICC(2,1) = 0.997 (95% CI [0.992, 0.999], absolute agreement, single measures). The Wilcoxon signed-rank test did not indicate a significant systematic difference between workflows (*p* = 0.329).

Bland–Altman analysis showed a mean bias of +2.88 mm^2^, with 95% limits of agreement from −16.31 to +22.08 mm^2^. These limits represented less than 8% of the mean contact area. This close correspondence at MIP supports the assumption that both workflows were anchored to a comparable functional origin. Therefore, subsequent differences observed during dynamic movements are more likely to reflect differences in kinematic reconstruction than major discrepancies in the initial intercuspal reference. This analysis should not be interpreted as an independent validation of the absolute hardware accuracy of either acquisition system.

### 3.3. Total Displacement Magnitude (SVD Norm)

Bland–Altman analysis showed minimal systematic bias and narrow limits of agreement for the total displacement norm across all three movement directions: bias = +0.010 mm (95% LoA: −0.076 to +0.096 mm) for left laterotrusion, bias = +0.006 mm (95% LoA: −0.147 to +0.160 mm) for right laterotrusion, and bias = +0.005 mm (95% LoA: −0.067 to +0.077 mm) for protrusion. Pearson correlations were also high (left r = 0.999; right r = 0.997; protrusion r = 0.999), but as correlation reflects association rather than agreement, these coefficients are reported descriptively only and are not used to support interchangeability between systems. Descriptive values are summarised in Table 2.

### 3.4. Directional Decomposition of the Displacement Vector

Although the two systems yielded closely matched scalar displacement magnitudes under the threshold-selection procedure, the directional decomposition of that displacement onto the three anatomical axes (X = mediolateral; Y = anteroposterior; Z = superoinferior) differed systematically across the dataset (Table 3). To preserve the independence of paired observations, the analysis was performed separately for each of the twelve movement × threshold conditions; each participant contributed at most one paired observation per cell.

During left laterotrusion, Zebris recorded significantly larger X (mediolateral) components than Medit at every threshold tested (all *p* ≤ 0.007; dz range +0.78 to +1.10, large effects), with mean bias increasing progressively from +0.186 mm at the 0.5 mm threshold to +0.606 mm at the 3.0 mm threshold. Conversely, Medit recorded significantly larger Y (anteroposterior) components at the 0.5, 1.0, and 3.0 mm thresholds (*p* = 0.049, 0.012, and < 0.001, respectively), and significantly larger Z (superoinferior) components at the 0.5, 1.0, and 2.0 mm thresholds (all *p* ≤ 0.035).

During right laterotrusion, the X-component bias in favour of Zebris was likewise significant at every threshold (all *p* ≤ 0.040; dz +0.56 to +0.92). Y- and Z-component differences were less consistent across thresholds: Medit recorded a significantly larger Z component at the 0.5 mm threshold (*p* = 0.021) and a significantly larger Y component at the 3.0 mm threshold (*p* = 0.021), while the remaining thresholds did not reach statistical significance, consistent with the small per-condition sample size.

During protrusion, the pattern changed in accordance with the predominantly sagittal nature of the movement. The X-component was significantly smaller in Zebris than in Medit at the 1.0 mm and 2.0 mm thresholds (*p* = 0.016 and 0.043, respectively). The Y-component was significantly larger in Zebris at the 0.5, 1.0, and 2.0 mm thresholds (all *p* ≤ 0.035). Z-component differences did not reach significance at any threshold during protrusion. The SVD reconstruction residual, interpreted only as an internal rigid-body fitting residual, remained significantly larger for Zebris than for Medit across every condition tested (all Wilcoxon *p* ≤ 0.050; r range 0.34–0.62).

Taken together, these condition-specific results indicate that inter-system differences in directional decomposition were reproducible across the displacement range, particularly for the X axis during laterotrusion and for the Y axis during protrusion. The statistical detection of Y- and Z-axis differences depended on the threshold and on the per-condition sample size. Effect sizes ranged from small (dz ≈ 0.24) to very large (dz ≈ 1.21), with the largest and most consistent effects observed for the X component during lateral excursions.

### 3.5. Limits of Agreement and Inter-System Correlation

Bland–Altman analysis was conducted to characterise the structure of inter-system differences beyond the central tendency. For the total norm, the bias was negligible across all three movements (|bias| ≤ 0.010 mm), and the 95% limits of agreement were narrow: half-width ≤ 0.080 mm in left laterotrusion, ≤0.077 mm in protrusion, and ≤0.154 mm in right laterotrusion. These limits remained within the predefined ±0.5 mm clinical reference margin, supporting the interpretation that the threshold-selection procedure yielded closely matched scalar displacement amplitudes between workflows. Pearson coefficients approached unity (r ≥ 0.997), but these coefficients are reported descriptively only and are not interpreted as evidence of agreement or interchangeability.

For the directional components, the limits of agreement were substantially wider. For example, the Y component in left laterotrusion ranged from −1.322 mm to +0.658 mm, while the Z component in the same movement ranged from −1.121 mm to +0.621 mm. This indicates that the inter-system differences in directional attribution were not only systematic, as reflected by the non-zero bias, but also heterogeneous between participants.

Pearson correlations between the decomposed component values were moderate to strong for the X and Z components (X: r = 0.81 left, 0.85 right; Z: r = 0.78 left, 0.90 right, 0.91 protrusion). Aggregated Bland–Altman bias, 95% limits of agreement, and correlation coefficients for the total displacement norm, directional components, and SVD residuals are reported descriptively in Table 4.

### 3.6. SVD Reconstruction Residual

The Frobenius-norm residual of the rigid-body SVD reconstruction, defined as the geometric distance remaining between the transformed and target marker configurations after optimal rigid alignment, was systematically smaller for Medit than for Zebris across the tested conditions (all Wilcoxon *p* ≤ 0.050; r range: 0.34–0.62).

When aggregated by movement, the mean Zebris residual was 0.024 mm during left laterotrusion (range 0.014–0.037 mm across thresholds), 0.046 mm during right laterotrusion (range 0.037–0.053 mm), and 0.024 mm during protrusion (range 0.015–0.029 mm). The corresponding Medit residual remained between 0.003 and 0.004 mm across all conditions, with markedly lower inter-subject variability.

In both workflows, SVD residuals remained well below the predefined 0.5 mm clinical reference margin used for occlusal measurement, supporting the internal numerical consistency of the rigid-body fitting procedure. However, these residuals should be interpreted as an internal measure of SVD fitting consistency rather than as direct evidence of superior clinical agreement. The lower Medit residuals indicate a tighter numerical fit of the marker configurations used by the algorithm, whereas the clinical meaning of this difference requires cautious interpretation.

### 3.7. Qualitative Heatmap Visualisation

**Figure 6:**
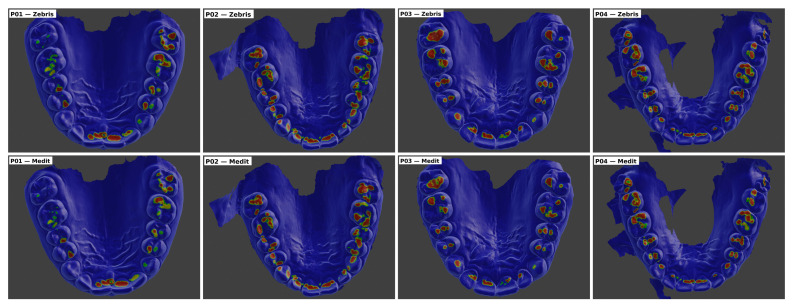
Validation of the common origin at maximum intercuspation. The visual similarity between workflows is consistent with the quantitative assessment of a comparable functional origin at MIP.

Qualitative heatmap visualisation was used to illustrate the clinical appearance of the kinematic differences identified in the quantitative analysis. Figure 6 shows the occlusal contact pattern at maximum intercuspation in four representative participants, comparing the Zebris-derived and Medit-derived reconstructions on the common STL/OBJ/PLY geometry. The visual similarity between the two rows was consistent with the quantitative assessment of a comparable functional origin between the two workflows. The colour scale ranged from red to yellow and green as the distance between the meshes increased.

**Figure 7:**
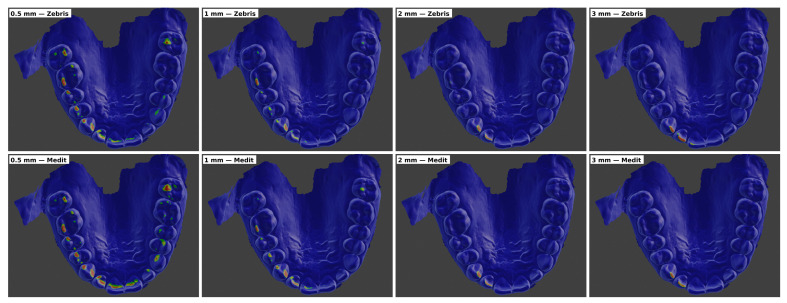
Evolution of the occlusal contact pattern during left laterotrusion in patient P02. Representative threshold-based comparison of Zebris-derived and Medit-derived reconstructions at 0.5, 1.0, 2.0, and 3.0 mm displacement from maximum intercuspation. Each column corresponds to one displacement threshold, with Zebris shown in the upper row and Medit in the lower row.

**Figure 8:**
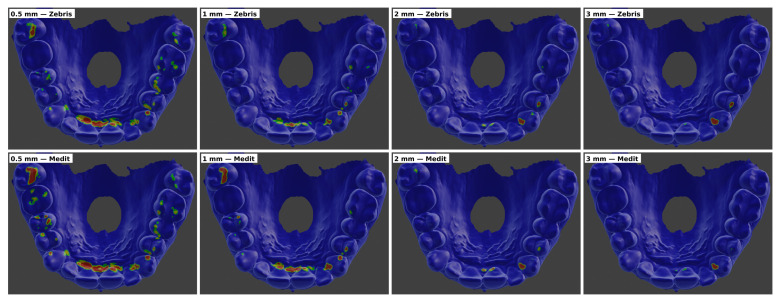
Evolution of the occlusal contact pattern during right laterotrusion in patient P05. Representative threshold-based comparison of Zebris-derived and Medit-derived reconstructions at 0.5, 1.0, 2.0, and 3.0 mm displacement from maximum intercuspation.

**Figure 9:**
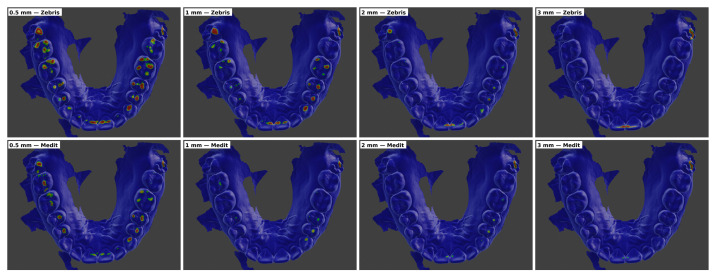
Evolution of the occlusal contact pattern during protrusion in patient P04. Representative threshold-based comparison of Zebris-derived and Medit-derived reconstructions at 0.5, 1.0, 2.0, and 3.0 mm displacement from maximum intercuspation.

Dynamic threshold-based heatmaps are presented in Figure 7, Figure 8 and Figure 9. Figure 7 illustrates left laterotrusion in patient P02 at the four predefined displacement thresholds of 0.5, 1.0, 2.0, and 3.0 mm. Figure 8 shows the corresponding sequence for right laterotrusion in patient P05, while Figure 9 presents the same threshold-based comparison during protrusion in patient P04.

In the laterotrusive movements shown in Figure 7 and Figure 8, the visual divergence between systems became more apparent as the displacement threshold increased. At equivalent total displacement thresholds, Medit-derived reconstructions generally showed a more pronounced reduction in close-proximity contacts, whereas Zebris-derived reconstructions tended to preserve a greater proportion of residual contacts in laterally displaced positions. This visual pattern was consistent with the component-wise SVD findings, in which Medit showed larger superoinferior components and Zebris showed larger mediolateral components during lateral excursions.

In protrusion, shown in Figure 9, the qualitative differences were less pronounced than during lateral movements and followed the predominantly sagittal nature of the movement. Overall, these visualisations provided a qualitative correlate of the main quantitative finding: the two workflows yielded closely matched scalar displacement amplitudes under the threshold-selection procedure, but differed in the spatial reconstruction of mandibular movement.

## 4. Discussion

### 4.1. Interpretation of Inter-System Agreement

The present study indicates that inter-system comparison of mandibular motion acquisition workflows should be interpreted at two distinct levels: scalar displacement magnitude and directional decomposition. Zebris JMA and Medit i700 yielded closely matched values for the total magnitude of mandibular displacement, calculated as the Euclidean norm of the SVD-derived translation vector. Across all movement directions and displacement thresholds, paired differences were small and remained well below the predefined ±0.5 mm clinical reference margin, indicating that the threshold-selection procedure identified similar scalar excursion amplitudes from maximum intercuspation.

When the same mandibular displacement was projected onto the anatomical axes, the two systems showed systematic differences. During laterotrusive movements, Zebris attributed a larger proportion of the displacement to the mediolateral axis, whereas Medit distributed a larger proportion of the same total displacement across the anteroposterior and superoinferior axes. During protrusion, the dominant component shifted toward the anteroposterior axis, consistent with the sagittal nature of the movement. The consistency of the directional bias across participants and movement conditions suggests that the observed differences were not purely random measurement noise, but reflected system-specific reconstruction behaviour.

The Zebris JMA system reconstructs mandibular kinematics through an external marker-based tracking workflow. Mandibular movement is derived from the trajectory of an infrared marker assembly attached to a paraocclusal mandibular fork and tracked relative to head-mounted infrared sensors. In contrast, the Medit i700 jaw motion module uses a scan-based workflow in which mandibular movement is reconstructed from dynamic interarch surface-tracking data. These fundamentally different acquisition principles may explain why the systems yielded closely matched scalar excursion amplitudes while distributing that displacement differently across the anatomical axes [17,18,19].

Importantly, the present findings do not demonstrate that one system is universally more accurate than the other. Without an independent gold-standard motion-tracking method, it is not possible to determine which reconstruction more closely represents true mandibular motion. The results should therefore be interpreted as evidence of different system-specific reconstruction patterns rather than as evidence of superiority of one acquisition method.

### 4.2. Clinical and Methodological Implications

When the variable of interest is the total scalar amplitude of mandibular excursion, Zebris JMA and Medit i700 yielded closely matched displacement values under the specific threshold-based frame-selection procedure used in this pilot study. This finding should not be interpreted as evidence of equivalence or interchangeability, because the total displacement norm was partly constrained by the analytical design.

Beyond the numerical agreement of the two systems, clinical feasibility is a relevant consideration for routine implementation. In the present pilot cohort, both Zebris JMA and Medit i700 achieved acquisition success rates exceeding 94% overall, and the difference between systems was not statistically significant at any threshold (McNemar’s exact *p* ≥ 0.500). The Medit system showed a marginally higher overall success rate (99.0% vs. 94.6%), driven by Zebris failures in one participant in whom rigid fixation of the mandibular paraocclusal fork could not be achieved. The Medit workflow avoids the use of an external cranial frame and paraocclusal fork, but may be affected by scanner-head fogging or condensation, temporary tracking loss, duplicated surfaces, inconsistent starting position, or incomplete dynamic capture during larger mandibular excursions.

Practical implementation also differs between the workflows. Zebris JMA requires a dedicated jaw-motion analysis system, placement of the cranial reference frame, identification of anatomical landmarks, calibration steps, and fixation of the mandibular paraocclusal fork. These procedures increase chair-side time and require operator training in the correct sequence of acquisition, fork adaptation, and fixation. They also introduce consumable costs for fixation materials and may reduce patient comfort, particularly in patients with deep overbite or limited interocclusal space where stable fork adaptation may be difficult.

Medit i700-based motion recording may be more accessible in practices where the intraoral scanner is already available, and it avoids the use of an external cranial frame and paraocclusal fork. However, this does not eliminate the need for acquisition control. Tracking loss, duplicated surfaces, inconsistent starting position, or incomplete excursions may affect the quality of the exported motion data and may not always be immediately evident to the operator. Therefore, both workflows require critical verification before clinical interpretation.

The open-source Blender/Blender4Dental framework reduces the software barrier to independent inspection of exported motion data and enables frame-level analysis that is not readily available in the commercial environments assessed in this study. However, open-source availability should not be interpreted as immediate chair-side scalability. The workflow still requires suitable hardware, familiarity with dental CAD/CAM concepts, Blender/Blender4Dental skills comparable to those required for advanced dental software environments, and understanding of mandibular kinematics. At its current stage, the framework should be considered a research and methodological tool rather than a production-ready clinical decision-support system.

When the clinical interpretation depends on the direction of movement, the two systems should not be considered fully interchangeable. Component-wise differences may influence the interpretation of laterotrusion, protrusion, immediate side shift, vertical separation, and dynamic occlusal relationships. It should be emphasized that the present threshold-based comparison does not imply that similar occlusal contacts could not occur at other frames or under different combinations of rotation and translation. Rather, the findings indicate that when the comparison is standardized to the same displacement from maximum intercuspation, the reconstructed spatial position of the mandible differs between systems. However, the present study did not directly test whether these component-wise differences change restoration morphology, occlusal adjustment decisions, or treatment planning. The framework supports future analysis of working- and non-working-side contacts through the definition of reference planes and side-specific contact-area outputs, but the clinical validation of these outputs was beyond the scope of this pilot investigation.

The SVD reconstruction residual was consistently lower for Medit than for Zebris. This indicates a tighter internal numerical fit of the marker configurations used by the SVD algorithm in the Medit-derived datasets. In the absence of an independent reference standard, residual differences may reflect differences in marker geometry, exported data structure, smoothing, tracking constraints, or manufacturer-specific XML encoding. The SVD residual therefore reflects the internal consistency of the rigid-body transformation for the available input data, not the absolute truth of mandibular motion [42,43].

### 4.3. Methodological Contribution of the Open-Source Framework

An important methodological contribution of this study is the development of an open-source analytical workflow for frame-level mandibular kinematic comparison. Commercial CAD/CAM platforms, such as Exocad 3.2 and Planmeca CAD Premium 3.0, allow mandibular motion to be visualised within digital dental workflows, but they generally provide limited access to the underlying frame-by-frame kinematic data, displacement components, threshold-specific positions, and independent analytical outputs [16,28,29].

Conversely, Blender and Blender4Dental provide an open and extensible three-dimensional environment, but they do not natively import XML jaw-motion files exported by Medit i700 or Zebris JMA. The custom Python 3.11 add-on developed in this study bridges this gap by importing vendor-specific XML files, identifying system-specific reference elements, reconstructing frame-by-frame mandibular motion using SVD-based rigid-body transformation, generating Blender keyframes, and enabling threshold-based analysis.

Within the limits of the implemented XML parsers and the tested acquisition systems, this framework transforms jaw-motion exports into an inspectable and reproducible analytical workflow. Instead of relying only on visual playback, the user can evaluate each recorded frame, quantify displacement magnitude, analyse directional components, identify clinically relevant thresholds, and compare different acquisition systems on the same anatomical geometry. The open-source nature of the publicly available GitHub release of the JMA XML Animator add-on (v1.0.0), together with the technical documentation provided in the Supplementary Materials, allows other researchers to inspect, reproduce, modify, and extend the framework for future validation studies.

### 4.4. Limitations

First, this was a pilot methodological study with a modest sample size of 17 participants. No formal sample-size calculation was performed because the study was designed as an exploratory paired-cohort comparison and software-workflow demonstration. Although the paired within-subject design reduced the influence of inter-individual anatomical variability, larger cohorts are required to confirm the generalisability of the observed inter-system patterns.

Second, the analysis was based on selected technically valid movement cycles rather than on averaged trajectories from multiple repeated cycles. Repeated recordings were performed when necessary to obtain valid acquisitions, but the final analysis did not quantify intra-session repeatability or inter-cycle variability. Therefore, the present study cannot fully separate measurement error from the natural biological variability of mandibular movement.

Third, all recordings were performed by a single operator. This improved procedural consistency but did not allow assessment of inter-operator reproducibility. The extent to which similar results would be obtained by different examiners remains to be investigated.

Fourth, no independent gold-standard or phantom-based motion-tracking reference with known translations and rotations was used. Therefore, the study compares the reconstruction behaviour of Zebris JMA and Medit i700 within the exported workflows, but cannot determine which system more closely represents true mandibular motion. Both acquisition systems also present workflow-specific sources of error. The Zebris workflow relies on an external reference frame and mandibular marker assembly, introducing potential registration and fixation-related errors. Optical tracking with the Medit i700 may be affected by scanner positioning and temporary tracking loss during larger mandibular movements [20,52].

Fifth, to address the possibility that the nearest-frame threshold-selection procedure may have introduced system-specific error due to the different native sampling frequencies of the two systems (Zebris 60 Hz; Medit 16–23 Hz), the absolute deviation between the selected-frame displacement and the target threshold was computed for every condition and is reported in Supplementary Table S1. Future iterations of the analytical framework could nevertheless implement temporal interpolation between recorded frames to further reduce this source of variability.

### 4.5. Future Directions

Future studies should include larger and more diverse cohorts, multiple operators, repeated acquisition cycles, formal repeatability testing, and phantom-based or reference-standard validation. They should also determine whether the observed directional discrepancies influence dynamic occlusal contact distribution, working- and non-working-side interpretation, restoration morphology, virtual articulator programming, or clinical decision-making. In addition, the working- and non-working-side contact-classification module could be applied to compare side-specific occlusal contact areas during laterotrusion, as well as anterior and posterior contacts during protrusion, to determine whether differences in displacement decomposition translate into measurable changes in occlusal contact patterns.

## 5. Conclusions

Within the limitations of this pilot methodological paired-cohort study, Zebris JMA and Medit i700 yielded closely matched scalar displacement values under the threshold-based frame-selection procedure, but differed systematically in the directional decomposition of mandibular motion. These findings should be interpreted as system-specific reconstruction behaviour within the exported XML workflows rather than as evidence of superiority, absolute accuracy, or direct interchangeability of either acquisition system.

The observed component-wise differences may be relevant for workflows in which movement direction influences dynamic occlusal interpretation, but the present study did not directly test whether these differences alter prosthodontic design, restoration morphology, occlusal adjustment, or treatment decisions. Therefore, broader clinical generalization requires validation in larger samples, with multiple operators, repeated cycles, phantom/reference-standard testing, and clinically relevant outcomes.

## Figures and Tables

**Table 1:** Number and proportion of participants in whom each system successfully reached the displacement threshold, per movement × threshold combination.

Movement	Threshold (mm)	Zebris (60 Hz)	Medit (16–23 Hz)	McNemar Exact *p*
Left lateral	0.5	16/17 (94.1%)	17/17 (100%)	1.00
Left lateral	1.0	16/17 (94.1%)	17/17 (100%)	1.00
Left lateral	2.0	16/17 (94.1%)	17/17 (100%)	1.00
Left lateral	3.0	15/17 (88.2%)	16/17 (94.1%)	1.00
Right lateral	0.5	16/17 (94.1%)	17/17 (100%)	1.00
Right lateral	1.0	16/17 (94.1%)	17/17 (100%)	1.00
Right lateral	2.0	16/17 (94.1%)	17/17 (100%)	1.00
Right lateral	3.0	16/17 (94.1%)	16/17 (94.1%)	1.00
Protrusion	0.5	17/17 (100%)	17/17 (100%)	—
Protrusion	1.0	17/17 (100%)	17/17 (100%)	—
Protrusion	2.0	17/17 (100%)	17/17 (100%)	—
Protrusion	3.0	15/17 (88.2%)	17/17 (100%)	0.500

Note. Each value indicates the number and percentage of participants in whom the corresponding system successfully reached the specified displacement threshold. McNemar’s exact test was applied to the paired success/failure proportions per condition; only discordant pairs contribute to the test. *p*-values are not reported for conditions in which no discordant pairs were observed.

**Table 2:** Descriptive statistics and paired mean differences for the total SVD displacement magnitude across all twelve movement × threshold conditions.

Movement	Threshold (mm)	*n*	Total Zebris (Mean ± SD)	Total Medit (Mean ± SD)	Mean Diff (Z − M)	95% CI
Left lateral	0.5	*16*	0.512 ± 0.022	0.500 ± 0.004	+0.012	[+0.001, +0.023]
Left lateral	1.0	*16*	1.001 ± 0.039	1.000 ± 0.004	+0.001	[−0.019, +0.020]
Left lateral	2.0	*16*	2.026 ± 0.070	2.001 ± 0.004	+0.025	[−0.009, +0.060]
Left lateral	3.0	*14*	3.001 ± 0.020	3.000 ± 0.004	+0.000	[−0.011, +0.012]
Right lateral	0.5	*16*	0.481 ± 0.062	0.500 ± 0.004	−0.020	[−0.054, +0.014]
Right lateral	1.0	*16*	1.007 ± 0.082	1.000 ± 0.004	+0.006	[−0.038, +0.051]
Right lateral	2.0	*16*	2.003 ± 0.079	2.000 ± 0.004	+0.003	[−0.040, +0.045]
Right lateral	3.0	*15*	3.038 ± 0.081	2.999 ± 0.005	+0.038	[−0.008, +0.084]
Protrusion	0.5	*17*	0.498 ± 0.027	0.500 ± 0.004	−0.002	[−0.016, +0.012]
Protrusion	1.0	*17*	1.005 ± 0.042	1.000 ± 0.004	+0.005	[−0.017, +0.026]
Protrusion	2.0	*17*	2.005 ± 0.041	2.001 ± 0.005	+0.004	[−0.018, +0.026]
Protrusion	3.0	*15*	3.015 ± 0.035	3.000 ± 0.004	+0.014	[−0.005, +0.034]

Note. Values are mean ± SD in mm. CI = confidence interval for the paired mean difference. Mean difference = Zebris − Medit. Sample size (*n*) varies by condition due to pairwise exclusion of trials in which one system did not reach the requested displacement threshold.

**Table 3:** Component-wise inter-system comparison of SVD displacement vector projections across movement × threshold conditions.

Movement	Threshold (mm)	Component	*n*	Zebris (M ± SD)	Medit (M ± SD)	Bias	95% CI for Bias	Test
Left lateral	0.5	*x (mediolateral)*	*16*	0.423 ± 0.090	0.237 ± 0.118	+0.186	[+0.103, +0.270]	paired *t*
Left lateral	0.5	*y (anteroposterior)*	*16*	0.121 ± 0.103	0.229 ± 0.138	−0.108	[−0.207, −0.009]	paired *t*
Left lateral	0.5	*z (superoinferior)*	*16*	0.181 ± 0.142	0.317 ± 0.104	−0.135	[−0.230, −0.041]	paired *t*
Left lateral	0.5	*SVD residual*	*16*	0.016 ± 0.019	0.003 ± 0.002	+0.013	[+0.003, +0.022]	Wilcoxon
Left lateral	1.0	*x (mediolateral)*	*16*	0.842 ± 0.149	0.503 ± 0.241	+0.338	[+0.187, +0.489]	paired *t*
Left lateral	1.0	*y (anteroposterior)*	*16*	0.182 ± 0.154	0.432 ± 0.297	−0.250	[−0.423, −0.077]	paired *t*
Left lateral	1.0	*z (superoinferior)*	*16*	0.401 ± 0.248	0.609 ± 0.238	−0.207	[−0.383, −0.032]	paired *t*
Left lateral	1.0	*SVD residual*	*16*	0.025 ± 0.029	0.004 ± 0.002	+0.021	[+0.007, +0.035]	Wilcoxon
Left lateral	2.0	*x (mediolateral)*	*16*	1.579 ± 0.412	1.168 ± 0.385	+0.412	[+0.151, +0.672]	paired *t*
Left lateral	2.0	*y (anteroposterior)*	*16*	0.490 ± 0.482	0.739 ± 0.558	−0.249	[−0.499, +0.002]	paired *t*
Left lateral	2.0	*z (superoinferior)*	*16*	0.897 ± 0.457	1.224 ± 0.419	−0.327	[−0.556, −0.098]	paired *t*
Left lateral	2.0	*SVD residual*	*16*	0.037 ± 0.065	0.003 ± 0.002	+0.034	[+0.002, +0.066]	Wilcoxon
Left lateral	3.0	*x (mediolateral)*	*14*	2.382 ± 0.631	1.775 ± 0.558	+0.606	[+0.235, +0.978]	paired *t*
Left lateral	3.0	*y (anteroposterior)*	*14*	0.453 ± 0.318	1.230 ± 0.572	−0.777	[−1.114, −0.440]	paired *t*
Left lateral	3.0	*z (superoinferior)*	*14*	1.503 ± 0.659	1.845 ± 0.606	−0.341	[−0.693, +0.010]	paired *t*

Note. Each row represents an independent paired comparison; each participant contributes one paired observation per condition. Values are mean ± SD in mm. Bias = mean of paired differences (Zebris − Medit). Test selection was data-driven: paired *t*-test when Shapiro–Wilk testing of paired differences yielded *p* > 0.05; Wilcoxon signed-rank test otherwise.

**Table 4:** Descriptive Bland–Altman parameters and inter-system Pearson correlations for SVD displacement components, aggregated per movement direction.

Movement	Component	Bias (mm)	Lower LoA (mm)	Upper LoA (mm)	Pearson r
Left lateral	*Total norm*	+0.010	−0.076	+0.096	0.999
Left lateral	*x (mediolateral)*	+0.379	−0.560	+1.317	0.814
Left lateral	*y (anteroposterior)*	−0.332	−1.322	+0.658	0.450
Left lateral	*z (superoinferior)*	−0.250	−1.121	+0.621	0.780
Left lateral	*SVD residual*	+0.020	−0.055	+0.095	0.027
Right lateral	*Total norm*	+0.006	−0.147	+0.160	0.997
Right lateral	*x (mediolateral)*	+0.275	−0.476	+1.026	0.851
Right lateral	*y (anteroposterior)*	−0.215	−1.172	+0.742	0.506
Right lateral	*z (superoinferior)*	−0.052	−0.643	+0.538	0.904
Right lateral	*SVD residual*	+0.043	−0.079	+0.164	0.267
Protrusion	*Total norm*	+0.005	−0.067	+0.077	0.999
Protrusion	*x (mediolateral)*	−0.135	−0.841	+0.571	0.320
Protrusion	*y (anteroposterior)*	+0.171	−0.582	+0.925	0.855
Protrusion	*z (superoinferior)*	−0.112	−0.770	+0.546	0.909
Protrusion	*SVD residual*	+0.021	−0.034	+0.075	0.013

Note. Aggregated values combine threshold-level observations within each movement direction and are presented descriptively.

## Data Availability

Data supporting the findings of this study are available from the corresponding author upon reasonable request. Raw patient STL/OBJ/PLY models and XML kinematic recordings are not publicly available due to privacy and ethical restrictions. The custom Python 3.11 add-on is available as open-source software through a public GitHub repository under an MIT License; repository information and usage documentation are provided in Supplementary Code S1, included in the Supplementary Files.

## Supplementary Materials

The following supporting information can be downloaded at: https://www.mdpi.com/article/10.3390/dj14070455/s1, Table S1: Absolute deviation between the selected-frame displacement and the target threshold, per system × movement × threshold combination. Table S2: Frame-selection deviation aggregated across all movements and thresholds, per system; Code S1: Public Repository Information for JMA XML Animator v1.0.0.

## Abbreviations

The following abbreviations are used in this manuscript:

3D	Three-dimensional
4D	Four-dimensional
CAD	Computer-aided design
CAM	Computer-aided manufacturing
CBCT	Cone-beam computed tomography
CI	Confidence interval
Hz	Hertz
ICC	Intraclass correlation coefficient
JMA	Jaw motion analyser
KD-tree	k-dimensional tree
LoA	Limits of agreement
LTS	Long-term support
M	Mean
MIP	Maximum intercuspation position
OBJ	Wavefront Object file format
PLY	Polygon File Format
RGB-D	Red–green–blue-depth
SD	Standard deviation
SPSS	Statistical Package for the Social Sciences
STL	Standard Tessellation Language
SVD	Singular value decomposition
XML	Extensible Markup Language
