# Supplementary material for: Comparative Analysis of Mandibular Kinematics Recorded with Zebris JMA and Medit i700 Using an Open-Source 3D Model-Based Framework
Source: Dent J (Basel). 2026 Jul 20;14(7):455. doi: 10.3390/dj14070455 (PMC13408630; doi:10.3390/dj14070455)
Supplement: Supplementary file 1 [file dentistry-14-00455-s001.zip › dentistry-4378468-supplementary.pdf]

Supplementary Table S1

Table S1. Absolute deviation between the selected-frame displacement and the target threshold, per system × movement × threshold combination.

Movement	Target threshold (mm)	System	n	Mean deviation (mm)	SD (mm)	Min (mm)	Max (mm)
Left lateral	0.5	Zebris	16	0.0161	0.0190	0.0000	0.0720
Left lateral	0.5	Medit	17	0.0032	0.0023	0.0000	0.0070
Left lateral	1.0	Zebris	16	0.0249	0.0291	0.0010	0.0960
Left lateral	1.0	Medit	17	0.0034	0.0022	0.0000	0.0070
Left lateral	2.0	Zebris	16	0.0369	0.0650	0.0010	0.2380
Left lateral	2.0	Medit	17	0.0032	0.0017	0.0010	0.0060
Left lateral	3.0	Zebris	15	0.0162	0.0135	0.0010	0.0470
Left lateral	3.0	Medit	16	0.0029	0.0023	0.0000	0.0070
Right lateral	0.5	Zebris	16	0.0377	0.0524	0.0010	0.2010
Right lateral	0.5	Medit	17	0.0039	0.0021	0.0000	0.0070
Right lateral	1.0	Zebris	16	0.0481	0.0658	0.0030	0.2320
Right lateral	1.0	Medit	17	0.0036	0.0021	0.0000	0.0070
Right lateral	2.0	Zebris	16	0.0454	0.0641	0.0030	0.2250
Right lateral	2.0	Medit	17	0.0030	0.0022	0.0000	0.0070
Right lateral	3.0	Zebris	16	0.0505	0.0697	0.0010	0.2250
Right lateral	3.0	Medit	16	0.0043	0.0019	0.0010	0.0070
Protrusion	0.5	Zebris	17	0.0151	0.0221	0.0000	0.0850
Protrusion	0.5	Medit	17	0.0036	0.0020	0.0000	0.0070
Protrusion	1.0	Zebris	17	0.0290	0.0299	0.0030	0.1190
Protrusion	1.0	Medit	17	0.0035	0.0027	0.0000	0.0070
Protrusion	2.0	Zebris	17	0.0276	0.0303	0.0000	0.1040
Protrusion	2.0	Medit	17	0.0041	0.0022	0.0000	0.0070
Protrusion	3.0	Zebris	15	0.0255	0.0279	0.0010	0.0910
Protrusion	3.0	Medit	17	0.0036	0.0018	0.0000	0.0060

Note. Deviation = |displacement of the selected frame - target threshold|. Values are in millimetres. *n* indicates the number of valid paired trials per condition.

Table S2. Frame-selection deviation aggregated across all movements and thresholds, per system.

System	n (observations)	Mean	SD	Median	Min	Max
Zebris (60 Hz)	193	0.0311	0.0457	0.0140	0.0000	0.2380
Medit (16–23 Hz)	202	0.0035	0.0021	0.0040	0.0000	0.0070

Note. Despite its lower nominal sampling frequency, the Medit system produced an order-of-magnitude smaller deviation than Zebris, suggesting that the Medit XML export contains frame data with finer effective resolution than the nominal acquisition rate would suggest. Both systems' deviations remain well below the 0.5 mm threshold of clinical relevance and substantially smaller than the inter-system biases observed for the directional displacement components (Tables 2 and 3 of the main manuscript).

Supplementary Code S1

Public Repository Information for JMA XML Animator v1.0.0

The custom Blender Python add-on developed in this study, JMA XML Animator v1.0.0, is available in a public GitHub repository:
https://github.com/radusmaximus/JMA-XML-Animator
The version corresponding to the present manuscript is archived as release v1.0.0:
https://github.com/radusmaximus/JMA-XML-Animator/releases/tag/v1.0.0
The repository includes the add-on code, documentation, software version requirements, installation instructions, and usage instructions. Raw clinical XML, STL, OBJ, or PLY datasets are not included because these files contain patient-related anatomical and kinematic information and are subject to privacy and ethical restrictions.
The software is provided for research and methodological validation purposes only and is not intended as a standalone clinical decision-support system.
